# Mammalian mitophagy – from *in vitro* molecules to *in vivo* models

**DOI:** 10.1111/febs.14336

**Published:** 2017-12-01

**Authors:** Catherine E. Rodger, Thomas G. McWilliams, Ian G. Ganley

**Affiliations:** ^1^ MRC Protein Phosphorylation and Ubiquitylation Unit School of Life Sciences University of Dundee UK

**Keywords:** autophagy, development, disease, metabolism, mitochondria, mitophagy, *mito*‐QC, mouse models, NIX, Parkin

## Abstract

The autophagic turnover of mitochondria, termed mitophagy, is thought to play an essential role in not only maintaining the health of the mitochondrial network but also that of the cell and organism as a whole. We have come a long way in identifying the molecular components required for mitophagy through extensive *in vitro* work and cell line characterisation, yet the physiological significance and context of these pathways remain largely unexplored. This is highlighted by the recent development of new mouse models that have revealed a striking level of variation in mitophagy, even under normal conditions. Here, we focus on programmed mitophagy and summarise our current understanding of why, how and where this takes place in mammals.

**Abbreviations**
AMPK: AMP‐activated protein kinase
CL: cardiolipin
DCT: distal convoluted tubule
Drp1: dynamin‐related protein 1
ER: endoplasmic reticulum
FUNDC1: FUN‐14 domain containing protein 1
IMM: inner mitochondrial membrane
iPSC: induced pluripotent stem cell
KO: knockout
LIR: LC3‐interacting region
MAM: mitochondria‐associated ER membrane
mTORC1: mammalian target of rapamycin complex 1
OMM: outer mitochondrial membrane
OXPHOS: oxidative phosphorylation
PCT: proximal convoluted tubule
PHB2: prohibitin 2
PINK1: PTEN‐induced putative kinase 1
PKA: protein kinase A
RGC: retinal ganglion cell
ULK1: UNC‐51 like kinase 1

## Introduction

**Figure 1:**
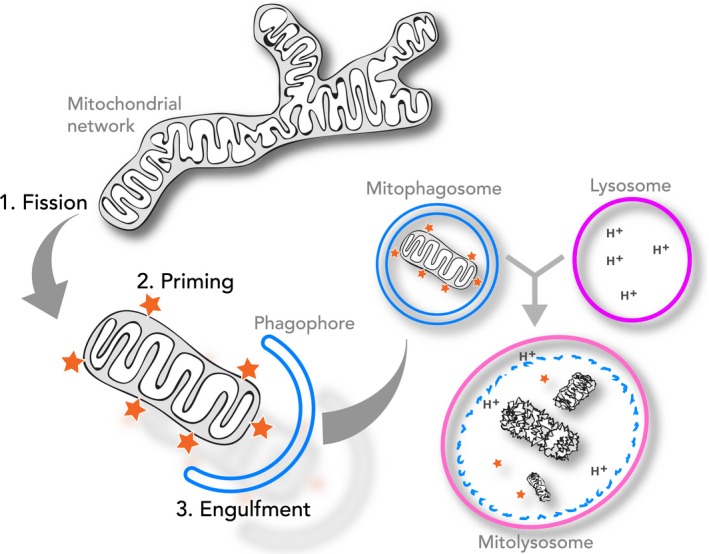
The requirements for mitophagy. Cartoon depicting the molecular events occurring during mitophagy. (1) Fission of the mitochondrial network into individual mitochondrion/mitochondrial fragments; (2) Marking of these to prime them for recognition by the autophagy machinery; (3) Formation of the autophagic phagophore to engulf the primed mitochondrion. Once engulfed, the mitochondrion‐containing autophagosome (mitophagosome) fuses with a lysosome to form the mitolysosomes where degradation and recycling occurs.

Mitochondrial homeostasis underpins cell survival. These multifunctional organelles are not only at the heart of cellular energy metabolism but also play key roles in many other processes, including calcium storage, iron metabolism and apoptosis. Maintaining a healthy mitochondrial population presents a huge cellular challenge and, as the primary sources of potentially hazardous reactive oxygen species and instigators of programmed cell death, failure to do so could have disastrous repercussions. This is especially important for the viability of long‐lived, postmitotic cells such as neurons and cardiomyocytes, where the preservation of mitochondria may underpin a healthy human lifespan. The accumulation of dysfunctional mitochondria has been tied to ageing and the progression of many human diseases, ranging from cancer to neurodegeneration. While cell signalling systems such as chaperones and proteases act to survey mitochondrial integrity, mitochondrial quality control also extends to the lysosome‐dependent autophagic turnover of entire organelles. During the selective destruction of damaged mitochondria, termed mitophagy, a portion of the mitochondrial network is gradually engulfed within a double‐membraned autophagosome, known as a mitophagosome. This eventually fuses with a compartment of the endolysosomal system, resulting in the proteolytic degradation of its mitochondrial cargo (Fig. 1). Notably, mitophagy is not the only lysosomal pathway implicated in mitochondrial turnover; for example, mitochondrial‐derived vesicles (MDVs) can also deliver damaged mitochondrial components to the lysosome and are thus also implicated in quality control 1. In this review, we will first discuss insights from cell‐based studies that have provoked great interest in the significance of mitophagy as a protective pathway during health and disease. We then describe our current mechanistic understanding of how it is coordinated. Finally, we discuss the advent of new *in vivo* mitophagy models that reveal the striking instances of mitophagy that occur under steady‐state physiological conditions.

## Reasons to trigger mitophagy

Multiple mitophagy pathways operate within the cell and fulfil various roles. Mitophagy is thought to perform a housekeeping role under steady‐state conditions, being responsible for the timely turnover of aged organelles. In addition, to combat the potential risks associated with dysfunctional mitochondria, mitophagy can also be rapidly enhanced as a protective mechanism to selectively remove damaged mitochondria and this has been extensively reviewed 2, 3, 4. More recently, it has become apparent that commitment of mitochondria to mitophagy‐dependent degradation is a core mechanism by which organelle quantity and/or functional characteristics may be adjusted. This is a variant of mitophagy, referred to as programmed mitophagy 5, described to occur independently of mitochondrial damage and is engaged in an array of developmental and physiological contexts.

Important insights into programmed mitophagy were first recorded by the Ney laboratory while studying erythrocyte differentiation and maturation. Erythrocytes rapidly develop from immature erythroblasts generated in the bone marrow over a period of 48–72 h 6. Erythroblasts are enucleated to form erythrocytes, before all remaining internal organelles and ribosomes are destroyed 6. During this process, the specific removal of mitochondria occurs via mitophagy 7, 8.

In addition to the role of mitophagy in erythrocyte development, mitophagy is also critical for the programmed of elimination of sperm‐contributed paternal mitochondria from fertilised oocytes during embryogenesis in *Caenorhabditis elegans* 9, 10, 11. However, it is not clear as to whether this is also true in mammals 12, 13, 14. Similarly, mitophagy is required for developmentally induced enterocyte cell death which occurs in the midgut of *Drosophila melanogaster* during metamorphosis 15.

Metabolic reprogramming is a characteristic of many differentiation pathways, and mitophagy has recently been described to influence this process. Programmed mitophagy has been shown to direct the metabolic transition which occurs during cardiomyocyte maturation. During the neonatal period, cardiomyocyte substrate preference switches from carbohydrates to fatty acids, and this is concurrent with the replacement of small, fetal mitochondria with morphologically distinct mature, adult mitochondria better suited to support the heightened energetic demand of mature cardiac muscle 16. Remarkably, mitophagy has been implicated in this maturation process: genetic disruption of mitophagy‐related signalling in mice results in fetal‐like mitochondria in adult animals, the development of progressive cardiomyopathy and death by 7–8 weeks of age 17.

Another example where developmentally induced mitochondrial changes occur in mitochondria is during the conversion of myoblasts into myotubes during skeletal muscle fibre development. Upon differentiation, when myoblasts ultimately fuse into multinucleated myofibres, their metabolic profile is reconfigured from glycolysis towards oxidative phosphorylation (OXPHOS) 18. Studies using cultured murine C2C12 myoblasts have shown that at this time, the mitochondrial population is initially cleared and then replenished with a structurally distinct network 19. Mitophagy is believed essential for this process and is, therefore, a potential key regulator of myogenesis 19.

Intriguingly, mitophagy itself can also promote glycolysis, a phenomenon first reported in cancer cells which can enhance mitophagy to upregulate glycolysis during prolonged mitotic arrest 20. A mitophagy‐triggered metabolic switch also occurs during the development of the embryonic mouse retina 21. In retinal ganglion cells (RGCs), the first retinal cell type to arise, differentiation occurs alongside a switch from OXPHOS to a highly glycolytic state. A block in mitophagy abrogates RGC differentiation and the associated bioenergetic changes, highlighting the pivotal role of mitophagy in this process 21.

Further elaborating on the fundamental importance of mitophagy, it has emerged as a key determinant for the maintenance of stem cell identity in embryonic stem cells, hematopoietic stem cells, and satellite cells, a population of muscle stem cells required for regeneration of skeletal muscle 22, 23, 24. Mitophagy is also an important feature of somatic cell reprogramming to induce pluripotent stem cells (iPSCs). Somatic cells and iPSCs have distinct metabolic identities, whereby somatic cells rely predominantly on OXPHOS and iPSCs are highly glycolytic. This metabolic shift is essential for reprogramming efficiency and is accompanied by a characteristic reduction in mitochondrial content 25. Critically, mitophagic activity accounts for the decrease in mitochondrial mass observed at the onset of somatic cell reprogramming and contributes to successful completion of iPSC generation 22, 26, 27.

## Mechanistic basics for mitophagy initiation

Mitophagy is a mechanistically elaborate process and critically depends on the regulated convergence of multiple signalling events. The basic requirements for mitophagy are summarised in Fig. 1. These include (a) fission of the mitochondrial network to produce a suitably sized mitochondrion that can be readily engulfed by the forming autophagosome; (b) priming of the mitochondrion for recognition by the autophagy machinery; and (c) engulfment of the marked mitochondrion by the forming autophagosome. Whether these three steps occur independently and sequentially, or in combination, remains to be determined.

### Fission

Mitochondrial dynamics are integral to mitochondrial function and the mechanics of mitophagy. Size constraints dictate that a typical mitochondrion cannot be accommodated within an autophagosome unless the network undergoes significant structural alteration. Mitochondrial fission refers to the severing of the mitochondrial network into smaller portions and is catalysed by the GTPase dynamin‐related protein 1 (Drp1) 28. At sites where endoplasmic reticulum (ER) tubules contact mitochondria, Drp1 is recruited from the cytosol by several adaptor proteins located at the surface of the organelle 29, 30, 31. Subsequent Drp1 oligomerisation and GTP hydrolysis leads to complete membrane constriction at fission sites 32. Importantly, fission commonly precedes and is necessary for mitophagy. Random fission events have been observed to generate two mitochondrial daughter units with uneven membrane potential 4. The fragments with diminished, unrecoverable membrane potential were unlikely to rejoin the mitochondrial network and more prone to turnover, thus providing a means to ensure the overall quality of the mitochondrial network 4. Conversely, it has also been shown that starvation‐induced elongation of the mitochondrial network spares mitochondria from degradation, enhancing OXPHOS potential during energy deprivation 33, 34. Enhanced mitochondrial connectivity has also been observed when mitochondrial OXPHOS is upregulated 35. Under these conditions, mitophagy is blocked as a direct consequence of defective mitochondrial fission 36.

It is essential to consider how the signals coordinating mitochondrial fission and mitophagy may be coupled. A direct role for the key mitophagy initiating kinase PTEN‐induced putative kinase 1 (PINK1), which is discussed in more detail below, has been proposed to play a role in this process. Following pharmacological agent‐induced depolarisation, PINK1 activity was shown to alleviate inhibitory protein kinase A (PKA)‐dependent Drp1 phosphorylation to promote fission 37. This is due to the displacement of PKA from AKAP1, a mitochondrial scaffolding protein that recruits PKA to the outer mitochondrial membrane (OMM), by an unknown mechanism. Another mitochondrial and mitophagy‐related protein, FUN‐14 domain containing protein 1 (FUNDC1), has been shown to instigate fission through direct physical association with Drp1. Wu *et al*. 38 demonstrated that a shift to hypoxic conditions induces the relocation of FUNDC1 to mitochondria‐associated ER membranes (MAMs), where it binds the MAM protein calnexin. Longer exposure to hypoxia reduces the interaction between FUNDC1 and calnexin, with the latter eventually being replaced with Drp1. Research by Chen *et al*. 39 showed that while Drp1 normally associates with the mitochondrial fusion factor OPA1, stress inducers such as selenite or carbonyl cyanide *p*‐(trifluoromethoxy)‐phenylhydrazon promote the binding of FUNDC1 to Drp1 instead.

The energy‐sensing kinase AMP‐activated protein kinase (AMPK) has strong links to mitochondrial function and has also been connected to mitochondrial dynamics 40. Upon its activation, either directly or through inhibition of the mitochondrial electron transport chain, AMPK phosphorylates mitochondrial fission factor (MFF) which increases mitochondrial Drp1 recruitment and subsequent fission. This is speculated to assist the mitophagic process.

Despite this, the mitophagy‐specific requirement of Drp1 has not been comprehensively established and alternate fission mechanisms may exist. Much work on the role of Drp1 in mitophagy has employed the use of mDivi1, a Drp1 small molecule inhibitor 41. Recent work suggests mDivi1 does not target Drp1 and instead functions as a mitochondrial complex I inhibitor 42. Additionally, there is mounting evidence for Drp1‐independent mitophagy 43, 44, 45. Remarkably, research from the Dorn laboratory revealed that mitophagy is actually promoted in the absence of Drp1 and inhibited in the absence of the fusion proteins Mfn1 and Mfn2 44. While adding to the complexity of the situation, this nonetheless attests to the importance of mitochondrial dynamics to mitophagy. Elegant work from Yamashita *et al*. 45 demonstrated an alternative scheme for mitochondrial fission upon mitophagy involving the autophagic machinery itself. They found that Drp1 is clearly absent from sites of autophagosome biogenesis at mitochondria and is dispensable for mitochondrial fission induced by a range of mitophagy stimuli. The autophagy machinery was first demonstrated to play a role in mitochondrial fission by the Chu Lab, which showed that siRNA of ATG7 or LC3 prevented mitochondrial fission in cells lacking PINK1 46. Interestingly, Drp1 was also implicated in this fission, implying that Drp1‐dependent and autophagy‐dependent fission may operate independently and co‐operatively. However, the contexts of these fission events are far from clear, and it remains to be determined if different mitophagy pathways have differing mitochondrial fission requirements.

### Priming mitochondria for mitophagy

The marking, or priming, of mitochondria for clearance is an essential step in mitophagy. Multiple factors, or receptors, have been identified which can orchestrate this by distinct mechanisms. Mitophagy receptors are central to mitochondrial priming and can be classified into two distinct groups; those which exist as integral mitochondrial proteins and those which indirectly associate with mitochondria through binding ubiquitylated mitochondrial proteins 47. All known protein mitophagy receptors possess a short, linear LC3‐interacting region (LIR) motif, which is responsible for their interaction with LC3/GABARAP proteins on the autophagosomal membrane. It consists of a core consensus sequence, as indicated by the single letter amino acid code: (W/F/Y)XX(L/I/V), where X can be any amino acid, and is often flanked by acidic residues 47.The LIR was first characterised in the ubiquitin‐binding protein p62/SQSTM1 as a critical determinant for the incorporation of polyubiquitylated aggregates into forming autophagosomes 48. Since this work, multiple LIR motif‐containing proteins have been identified and mediate the autophagy of various cargoes, in addition to mitochondria 49. Whether these receptors rely solely on their LIR motifs for cargo incorporation is not clear, but the receptors themselves are critical.

#### PINK1/Parkin‐mediated mitophagy

The most studied mitophagy pathway to date is mediated by the products of two genes that are mutated in autosomal recessive forms of Parkinson's disease (PD) 50, 51. Specifically, the mitochondrial‐associated protein kinase PINK1 and cytosolic RBR E3‐ubiquitin ligase Parkin play critical roles in damage‐induced mitophagy (see 3, 52, 53 for the landmark papers and reviewed in 54). Under basal or steady‐state conditions, cellular PINK1 levels remain low due to constitutive processing at mitochondria prior to subsequent N‐end rule degradation 55. PINK1 acts as a molecular sensor of mitochondrial damage; mitochondrial membrane depolarisation inhibits PINK1 cleavage and degradation, leading to its accumulation on the OMM. PINK1 stabilisation is associated with enhanced kinase activity, dimerisation and autophosphorylation 56, 57, 58, 59. The subsequent recruitment of cytosolic Parkin to mitochondria is dependent on PINK1 activation 52, 59, 60, 61, 62. Parkin exists in an auto‐inhibited conformation mediated by intra‐molecular interactions within its UBL domain 63, 64. PINK1‐mediated phosphorylation of both Parkin 57, 65 and ubiquitin at their respective serine 65 residues is necessary for full Parkin activation 66, 67, 68. Parkin catalyses the *de novo* conjugation and elongation of pre‐existing ubiquitin chains onto a host of OMM proteins, which are predicted to serve as PINK1 substrates 66, 67, 68. This increase in phospho‐ubiquitin chains on depolarised mitochondria constitutes a feed‐forward amplification loop that results in the completion of mitophagy 69, 70, 71. It is important to note that Parkin is not the only E3 ubiquitin ligase that has been implicated in mitophagy, with ligases such as MUL1 possibly performing a similar role 14. Additionally, ubiquitylation itself is reversible and deubiquitylases have thus been shown to be negative regulators of mitophagy 72.

Ubiquitylation of OMM proteins enables the recruitment of mitophagy receptors which possess a ubiquitin‐binding domain in addition to a LIR motif. These include p62/SQSTM1, OPTN, NDP52, TAX1BP1 and NBR1 73. Utilisation of genome‐editing technology has revealed that NDP52 and OPTN are the essential receptors for mitophagy, with a lesser contribution from TAX1BP1, while NBR1 and p62 are dispensable 74, 75. Surprisingly, OPTN and NDP52 are recruited to mitochondria even in the absence of Parkin and mitochondrial damage, and this is sufficient to induce mitophagy, albeit at a very low level 74.

Recently, the Levine laboratory identified prohibitin 2 (PHB2) as the first inner mitochondrial membrane (IMM) mitophagy receptor, which they found to be required for Parkin‐dependent mitophagy 76. In Parkin‐overexpressing cells subject to mitochondrial damaging agents, proteasomal degradation of the OMM makes IMM components accessible. Successful mitophagy relies on an interaction between PHB2 and LC3 via its LIR 76. Taken together, this suggests that a combination of mitophagy receptors is required for efficient mitochondrial priming.

Although much of the experimental evidence obtained to date supports the notion that PINK1 and Parkin are involved in damage‐induced mitophagy, this pathway has also been implicated in the regulation of programmed mitophagy. Indeed, the previously mentioned mitophagy that occurs during cardiomyocyte development is described to be PINK1/Parkin‐dependent 17.

#### BNIP3 and NIX/BNIP3L

Collectively, the findings amassed to date undoubtedly substantiate the importance of PINK1 and Parkin as central regulators of mitophagy. However, multiple other mitophagy pathways exist in addition. Indeed, our overall understanding of mitophagy has been propelled by evidence demonstrating that mitophagy is also mediated by receptors constitutively localised to mitochondria in a PINK1/Parkin‐independent manner. The hypoxia‐inducible proteins BNIP3L/NIX and its closely related homologue BNIP3 were the first mammalian mitophagy receptors identified 7, 8, 77. They were first characterised as pro‐apoptotic BH3‐only Bcl‐2 family proteins, both localised to the OMM by way of a C‐terminal transmembrane domain 78. Both NIX and BNIP3 contain identical LIRs at their N termini which facilitate interaction with autophagosomal LC3/GABARAP proteins 79. Both NIX and BNIP3 have been implicated in mitophagy; however, currently NIX is the most widely studied of the two proteins.

The mitophagic role of NIX was first illustrated in the context of erythropoiesis. NIX, which is transcriptionally upregulated during erythroid differentiation, is essential for mitochondrial clearance via mitophagy during the terminal stages of this process 7, 8, 80. Strikingly, in erythrocytes from NIX‐deficient mice, mitochondria fail to be incorporated into autophagosomes, implying that NIX is critical for recruitment 7, 8. Ablation of NIX interrupts erythroid maturation, resulting in anaemia, reticulocytosis and erythroid‐myeloid hyperplasia.

Besides erythroid differentiation, NIX is also the receptor required for mitophagy during somatic cell reprogramming to iPSCs and for RGC differentiation 21, 26. During RGC differentiation, local hypoxia transcriptionally induces NIX, accumulation of which triggers both mitochondrial removal and activation of glycolysis. Retinas from mice lacking NIX retain their mitochondria and exhibit fewer RGCs 21.

NIX is also involved in mitophagy induced when the demand for mitochondrial OXPHOS is high 81. In these circumstances, the small GTPase Rheb is recruited to the OMM where it is proposed to stimulate mitophagy through binding NIX and recruiting LC3 81. Accelerated rejuvenation of the mitochondrial pool is believed to contribute to increased OXPHOS efficiency.

A role for BNIP3 and mitophagy has been implicated in cancer development. In a mouse model of mammary tumorigenesis, loss of BNIP3 resulted in reduced mitophagy, the accumulation of functionally impaired mitochondria, elevated ROS production and consequent stabilisation of HIF1α 82. Increased HIF1α target gene expression favoured angiogenesis and bioenergetic changes associated with Warburg metabolism that ultimately promoted invasiveness and metastasis.

Although BNIP3 and NIX are primarily under transcriptional control, their mitophagic activity is also activated by post‐translational modification. Phosphorylation by an unknown kinase adjacent to the LIR of NIX and BNIP3 strengthens the association with LC3 to enhance mitophagy 83, 84.

#### FUNDC1

FUNDC1, an OMM‐spanning protein with an N‐terminal LIR motif, is another mitophagy receptor which functions independently of the PINK1/Parkin axis, and is responsible for hypoxia‐induced mitophagy 85. Short‐term regulation of FUNDC1 is carefully controlled by its phosphorylation status. In unstressed conditions, FUNDC1 is phosphorylated by Src kinase at tyrosine 18 and CK2 at serine 13, blocking its interaction with LC3 85, 86. PGAM5 is a mitochondrially localised phosphatase capable of dephosphorylating FUNDC1 at serine 13 86. Its activity is usually held in check through inhibitory interaction with Bcl‐xL; however, Bcl‐xL is degraded upon hypoxic stress, thereby enhancing PGAM5 phosphatase activity and promoting the targeting of mitochondria to LC3‐decorated autophagosomes for engulfment 87. The interaction between FUNDC1 and LC3 can be further strengthened following UNC‐51‐like kinase 1 (ULK1)‐dependent phosphorylation of FUNDC1 at serine 17 88. Beyond phosphorylation, ubiquitylation has also been implicated as a FUNDC1 control mechanism 89. The OMM‐localised E3 ligase MARCH5 can ubiquitylate FUNDC1 at the early stages of hypoxia, before FUNDC1 dephosphorylation, to prevent excessive mitophagy 89.

#### Bcl2L13

Bcl2L13, first identified as a pro‐apoptotic and atypical Bcl2 family member 90, is another OMM‐anchored mitophagy receptor which can recruit LC3/GABARAP proteins to mitochondria during mitophagy 43. Interestingly, this study showed that Bcl2L13 is multifunctional with respect to mitophagy as it mediates both mitochondrial fission and mitophagy upon mitochondrial damage. While the BH2 domains of Bcl2L13 were required for mitochondrial fragmentation, mutation of its internal LIR motif precludes association with LC3. Thus, both BH2 and LIR domains appear vital for mitophagy.

#### FKBP8

FKBP8/FKBP38 is a unique member of the FK506 family of binding proteins, distinguished by the fact that it possesses Ca^2+^/calmodulin‐activated peptidyl‐prolyl cis‐trans isomerase activity. It has been linked to multiple cellular processes including apoptosis and regulation of mTOR 91. Among the known mitophagy receptors in the OMM, FKBP8 is unique in that it itself avoids mitophagic turnover by rerouting to the ER 92, 93. While solid evidence of endogenous FKBP8 participating in mitophagy is lacking, it has been demonstrated that Parkin‐independent mitophagy ensues upon overexpression of both LC3 and FKBP8. The exact mechanism by which FKBP8 escapes degradation is unclear, as are the reasons for this intriguing behaviour, although it depends on a number of basic residues within its C‐terminal sequence 92, 93.

#### Cardiolipin

Protein receptors are not the only mode of mitophagy priming. The mitochondrially localised and unique anionic phospholipid cardiolipin (CL) can also act as a receptor. CL is unusual in that it is almost exclusively confined to the IMM. Here, it is an important regulator of mitochondrial bioenergetics, as association between CL and electron transport chain components is required for their optimal activity 94. Exposure to a range of mitophagic stimuli has also been shown to cause translocation of CL to the OMM, where it is free to interact with the N‐terminal of LC3 to facilitate capture of the mitochondrion into the forming autophagosome 95. The translocation of CL to the OMM was recently shown to require NDPK‐D, an intermembrane space‐localised nucleoside diphosphate kinase, which binds CL following mitophagy induction 96. CL has also been shown to regulate mitochondrial fission and fusion through recruitment and activation of Drp1 and OPA1; therefore, it may play multiple roles in mitophagy 97, 98, 99. Additionally, other lipids may also play an important role in regulating mitophagy 100.

While we have discussed the various mitochondrial receptors individually, it is possible that they do not operate in isolation. For example, there is evidence to support the idea that crosstalk exists between PINK1/Parkin‐mediated mitophagy and OMM receptor‐mediated mitophagy pathways 101, 102, 103. However, it has been clearly demonstrated that NIX‐mediated mitophagy is active even in the absence of a functional PINK1/Parkin pathway, as overexpression of NIX in human fibroblasts, isolated from patients with Parkin or PINK1‐related early‐onset PD, was capable of restoring carbonyl cyanide m‐chlorophenylhydrazone‐induced mitophagy 104. While potential crosstalk cannot be ruled out, its nature and regulation require further investigation.

### Mitochondrial engulfment by the autophagy machinery

Coincident with marking mitochondria for degradation, the autophagy machinery must be activated to drive formation of the engulfing autophagosome. While the relevant upstream signalling events are well defined in the context of classical starvation‐induced autophagy (reviewed in 105, 106, 107), the sensing mechanisms and signals that set the stage for targeted mitophagy induction are less clear.

#### Autophagy activating signals

At the helm of the canonical starvation‐induced autophagy pathway is the ULK1 protein kinase complex (comprising ULK1, ATG13, FIP200 and ATG101). ULK1 complex activation is essential for the early stages of autophagosome biogenesis 105 and as such has been implicated in both programmed and damage‐induced mitophagy. With respect to the former, ULK1 knockout (KO) mice display impaired removal during reticulocyte development 108, 109. As for the latter, ULK1 has also been shown to be critical for mitochondrial clearance in the PINK1/Parkin‐mediated pathway 74, 110 as well as during hypoxia 88.

##### mTORC1‐ULK1

The major negative regulator of canonical autophagy is the nutrient‐sensing protein kinase complex mammalian target of rapamycin complex 1 (mTORC1). In nutrient replete conditions, active mTORC1 restrains autophagic induction through inhibitory phosphorylation of ULK1 105. Repression of mTORC1 and dephosphorylation of ULK1 is essential for the activation of autophagy. In terms of mitophagy, there has been only limited analysis of the mTORC1 signalling pathway. In cybrid cells depleted of mtDNA, but not in wild‐type counterparts, mitophagy is provoked using the mTORC1 inhibitor rapamycin in conjunction with mitochondrial depolarisation 111. In line with this, during neurogenesis, rapamycin treatment has also been observed to induce mitophagy and promote RGC differentiation 21. Conflictingly, mitophagy mediated by Rheb under OXPHOS‐dependent conditions occurs in the absence of mTORC1 deactivation 81. Further work is obviously needed to clarify the role of the ULK1‐mTORC1 axis during mitophagy stimulation.

##### AMPK‐ULK1

Along with mTORC1, AMPK plays a significant role in regulating ULK1 activity in general autophagy. Multiple studies have identified AMPK‐dependent phosphorylation sites within ULK1 112, 113, 114, 115, 116. However, with varying overlap in sites identified between studies, and both activating and inhibitory effects reported, the relationship between AMPK and ULK1 remains obscure.

Consistent with a functional connection between AMPK and ULK1, mitochondrial removal is suppressed during the terminal differentiation of erythrocytes from AMPKα1 KO mice 117. It has also been reported that upon hypoxia, AMPK directly phosphorylates ULK1 at S555 118. This triggers ULK1 translocation to mitochondria and is critical for mitophagic induction. As mentioned previously, AMPK has been shown to regulate mitochondrial fission 40 and this opens up the possibility that AMPK coordinates multiple steps of mitophagy induction.

#### Recruitment of the autophagy machinery by mitophagy receptors

The long‐held notion is that a key function of the mitophagy receptors is to recruit a forming autophagosome to the mitochondrion via their ability to bind the autophagosomal LC3/GABARAP proteins. However, this may not be the only role the receptors play, with evidence suggesting that they may function earlier at the autophagosome initiation stage. Autophagosome generation is believed to be initiated at the mitochondrion itself and mitochondrial recruitment of upstream autophagy factors, including ULK1, DFCP1 and ATG14, has been shown to occur in the absence of LC3 45, 110. Indeed, during mitophagy, there does not seem to be an absolute requirement for LC3/GABARAP proteins in autophagosome formation 119, 120. Instead, recent observations suggest that LC3 functions at the autophagosome‐lysosome fusion step and can mediate the subsequent degradation of the inner autophagosomal membrane 119, 120. Therefore, receptor interaction with the autophagy signalling machinery may be key.

In the case of PINK1/Parkin‐dependent mitophagy, recruitment of the autophagy machinery to mitochondria has been reported to occur downstream of mitochondrial priming. Although ULK1 regulation by AMPK and mTORC1 occurs independently to the core mitophagy receptors OPTN and NDP52, they are thought to be indispensable for ULK1 recruitment to mitochondria 74. The subsequent recruitment of LC3, WIPI1 and DFCP1 is thus also reliant on both OPTN and NDP52.

Notably, FUNDC1 was also proposed to be a mitochondrial receptor for ULK1 88. In this hypoxia‐induced mitophagy pathway, ULK1 translocation to mitochondria is dependent on FUNDC1, and their association is necessary to accomplish mitochondrial degradation 88.

As is summarised in Table 1, a complex molecular picture of mitophagy is emerging with multiple mechanisms proposed to regulate both damage‐induced and programmed mitophagy. However, most of these mechanistic insights have been derived from cultured cell lines treated under nonphysiological conditions. Therefore, the major future challenge is to determine when and where these pathways operate *in vivo*.

**Table 1:** Proteins involved in the different stages of mitophagy induction

Protein	Function	References
Stage of mitophagy		
Fission		
Drp1	Dynamin‐related GTPase	37, 38, 39, 40, 46
PINK1	Mitochondrial protein kinase	37
PKA	Multifunctional protein kinase	37
AKAP	Mitochondrial PKA scaffolding protein	37
AMPK	Energy‐sensing protein kinase	40
MFF	DRP1‐interacting mitochondrial protein	40
FUNDC1	Mitophagy receptor	38, 39
BCL2L13	Mitophagy receptor	43
FIP200/ATG14/WIPI2/ATG7	Autophagy initiation machinery	45, 46
CL	Mitochondrial phospholipid	96, 97, 98
Priming		
PINK1	Mitochondrial protein kinase	52, 53, 54
Parkin	E3 ubiquitin ligase	3, 54
Phospho‐ubiquitin	PINK1 substrate	66, 67, 68
p62	Ubiquitin‐binding autophagy/mitophagy receptor	73, 74, 75
OPTN	Ubiquitin‐binding autophagy/mitophagy receptor	73, 74, 75
NDP52	Ubiquitin‐binding autophagy/mitophagy receptor	73, 74, 75
NBR1	Ubiquitin‐binding autophagy/mitophagy receptor	73, 74
TAX1BP1	Ubiquitin‐binding autophagy/mitophagy receptor	73, 74
PHB2	Mitophagy receptor	76
NIX	Mitophagy receptor	7, 8, 21, 26, 81
Rheb	mTOR‐activating GTPase	81
BNIP3	Mitophagy receptor	82, 83
FUNDC1	Mitophagy receptor	85, 86, 87, 88, 89
PGAM5	Mitochondrial protein phosphatase	86, 87
CK2	Protein kinase	86
Src	Tyrosine protein kinase	85, 86
MARCH5	Mitochondrial E3 ubiquitin ligase	89
ULK1	Autophagy‐initiating protein kinase	88
BCL2L13	Mitophagy receptor	43
FKBP8	Mitophagy receptor	92, 93
CL	Mitochondrial phospholipid	95, 96
Engulfment		
ULK1	Autophagy‐initiating protein kinase	73, 74, 88, 108, 109, 110
mTORC1	Nutrient‐sensing protein kinase	21, 81, 111
AMPK	Energy‐sensing protein kinase	112, 113, 114, 115, 116
OPTN	Mitophagy receptor	73
NDP52	Mitophagy receptor	73
FUNDC1	Mitophagy receptor	88

## Investigating mammalian mitophagy *in vivo*

**Figure 2:**
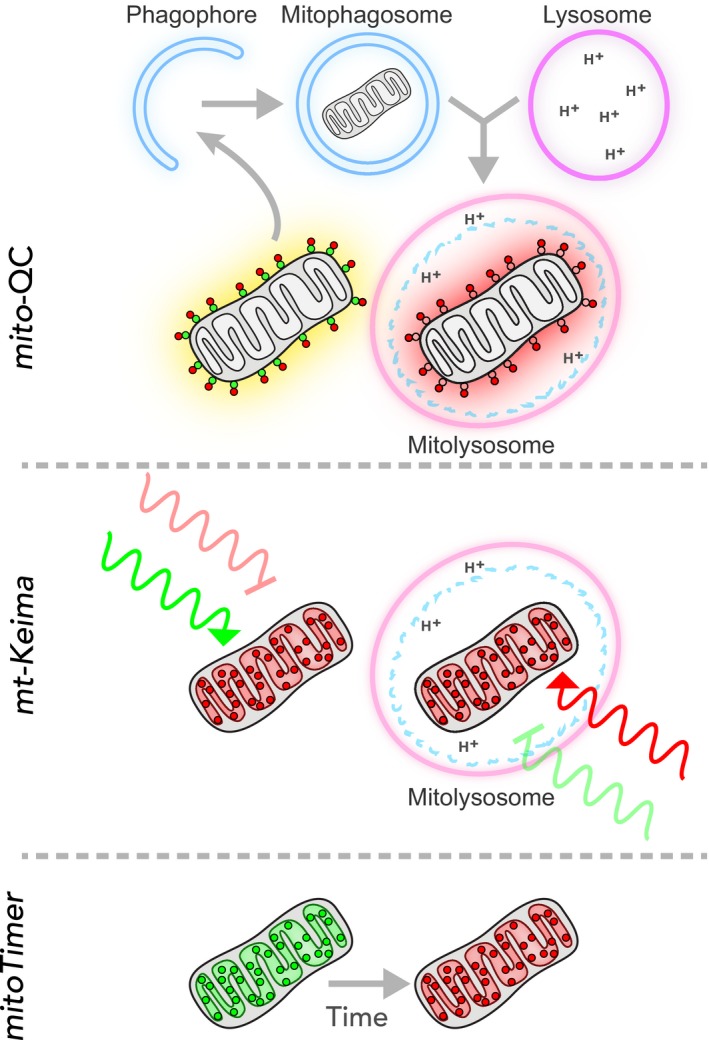
Mitophagy reporters. Schematic detailing the different reporters used to monitor mitophagy. In *mito*‐QC, a tandem mCherry‐GFP tag is targeted to the OMM. Under normal cytosolic conditions the mitochondria display both mCherry and GFP fluorescence. However, upon mitophagy, the low pH of the mitochondrion‐containing lysosome (mitolysosome) is sufficient to quench the GFP, but not mCherry, signal. In mt‐Keima, monomeric Keima protein is targeted to the mitochondrial matrix. Under normal cytosolic conditions, mt‐Keima is excited by light peaking at 400 nm, whereas this increases to 568 nm under lysosomal conditions, i.e. upon mitophagy. mt‐Keima's emission spectra remains the same, at 620 nm, regardless of this pH shift. For MitoTimer, the fluorophore DsRed1‐E5 was targeted to the mitochondrial matrix. Though strictly not designed to monitor mitophagy, it provides a powerful way in which to monitor the age of mitochondria as fluorescence shifts from green to red over a period of 48 h.

It has proved challenging to evaluate the physiological significance of numerous *in vitro* observations pertaining to mitophagy. This was due to a distinct lack of tools that facilitate the reliable detection of mammalian mitophagy *in vivo*. Recently, two mouse models have been described that utilise fluorescent reporter systems to enable the detection of mitophagy *in vivo* (*mito*‐QC reported by 121 and mt‐Keima reported by 122). These models exploit the acid labile properties of fluorescent reporter proteins to provide end‐point readouts of mitophagy *in vivo*. As such, the readout of mitophagy in the context of the current *in vivo* reporters is defined in terms of mitochondrial delivery to the acidic microenvironment of the lysosome. Under these conditions, the acidity either quenches the GFP signal (in the case of *mito*‐QC) or shifts the excitation wavelength (in the case of mt‐Keima, see Fig. 2). Both reporters faithfully monitor mitophagy and are ubiquitously expressed, though from different loci (*Rosa26* for *mito*‐QC and *Hip11* for mt‐Keima). As the reporters monitor the end‐point of mitophagy, they do not distinguish between distinct mitophagic pathways. However, the reporters localise to different mitochondrial compartments: mitochondrial outer membrane localisation for *mito*‐QC versus matrix localisation for mt‐Keima. This localisation is important as it may help distinguish between the previously mentioned MDV pathway, which represents an alternate mode of mitochondrial protein delivery to lysosomes 1. In contrast to mt‐Keima, the MOM topology of *mito*‐QC means that it will never be delivered to the lysosomal lumen through the more classical transport mechanism of the MDV pathway – hence, the mCherry‐only signal cannot be a consequence of this. Another model termed MitoTimer has also been described 123; however, this is an inducible model designed to monitor mitochondrial biogenesis over time, as well as mitophagy. Differences between these models are illustrated in Fig. 2, and these have also been recently reviewed 124. Regardless, these collective advances have transformed the landscape of mammalian mitophagy, enabling the interrogation of mitophagy and mitochondrial homeostasis in a variety of physiological contexts 54, 125.

### Basal mitophagy as a pervasive yet heterogeneous process in tissues

Perhaps, the most surprising conceptual discovery arising from these models is converging data that demonstrate the basal nature of mitophagy *in vivo*. Although differences in these models are well described 121, both studies report highly pervasive instances of mitophagy under normal everyday conditions. This is in stark contrast to the still widely held perception of mitophagy as merely an ‘activated autophagy response’ triggered to mitigate mitochondrial meltdown. This raises several important and unanswered questions: what are the tissue‐specific regulators of basal mitophagy *in vivo*? Do any of the aforementioned effectors of stimulus‐induced mitophagy modulate basal homeostatic mitophagy *in vivo*? Does disruption of these basal pathways have any pathophysiological consequences?

### Basal mitophagy and metabolic context

Although both mitophagy models revealed an extensive amount of basal mitophagy, studies using *mito*‐QC have highlighted the striking heterogeneity in mitophagy levels between defined cellular subsets *in vivo*. Thus, while all organ systems exhibit some degree of mitochondrial turnover, not all cells exhibit comparable levels of mitophagy *in vivo*. This is particularly evident in organ systems of high metabolic demand such as the nervous system, the renal system and cardiovascular system. In the brain, Sun *et al*. 122 were able to demonstrate regionalised instances of mitophagy in unlabelled tissue. However, given the breathtaking morphological complexity and diversity of neural subtypes, a more precise analysis is required to accurately interpret such observations in anisotropic tissue. Using *mito*‐QC combined with immunohistochemistry and high‐resolution microscopy, McWilliams *et al*. 121 showed specifically that labelled cerebellar Purkinje neurons exhibit robust levels of mitophagy, which was largely enriched within the somata. Future work will be essential to determine the spatial profile and regulation of mitophagy in different neuronal and nonneuronal subtypes. Furthermore, could basal mitophagy be elevated or diminished in genetic models of neurodegenerative disease? Interestingly, Sun *et al*. demonstrated diminished mitophagy in the context of Huntingtin overexpression 122. PINK1/Parkin signalling has gained much attention in stimulated mitophagy pathways; however, we have no idea if this signalling influences basal mitophagy *in vivo*. Interrogating this signalling is also troublesome, due to lack of reliable tools available to robustly detect endogenous PINK1 protein at basal levels *in vivo*. The requirement of endogenous Parkin under conditions of chronic mitochondrial stress was elegantly demonstrated by the Youle laboratory, who observed progressive age‐dependent DA neurodegeneration in Parkin KO mice crossed with the mutator (*Polg* ^D257A^) mouse model 126. However, it remains unclear if dysregulated mitophagy lies at the root of pathology in the double‐mutant mice. Understanding mitophagy in a mouse model exhibiting symptomatic neurodegeneration will prove vital to the interpretation of dysregulated mitophagy and its contribution to the progression of diseases such as PD.

**Figure 3:**
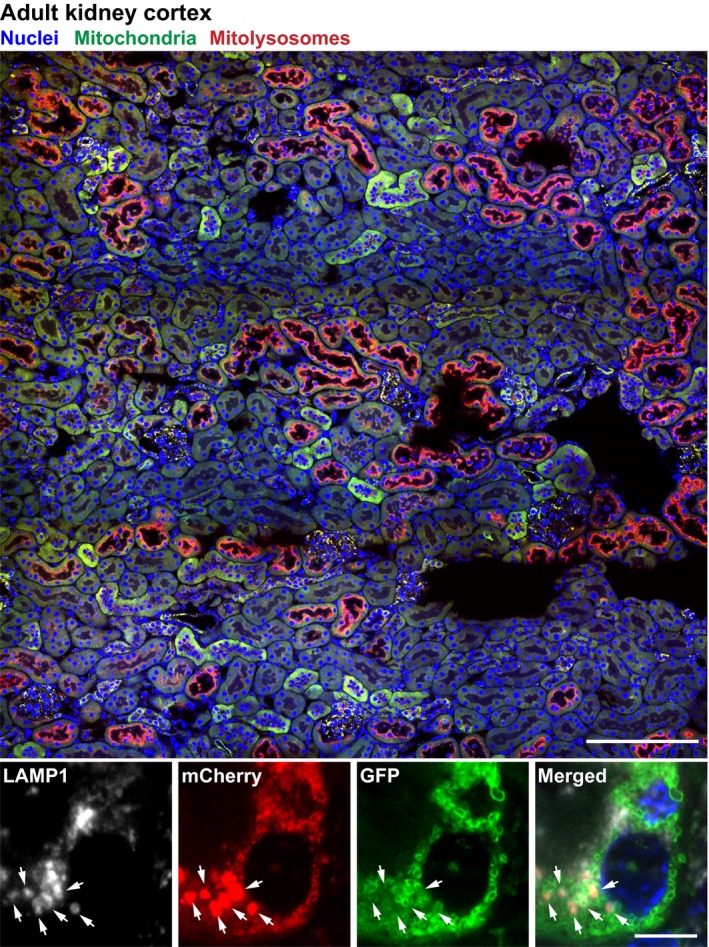
Mitophagy in the cortex of the adult kidney. Upper panels show a confocal micrograph (tile scan) of a section of adult kidney cortex from the *mito*‐QC reporter mouse under basal conditions. Note the distinct and polarised degree of mitophagy, as visualised by the ring‐like mCherry (red) signals, in a subset of kidney tubules (PCTs). Scale bar, 200 μm. Lower panels show a magnified *mito*‐QC PCT cell detailing the GFP and mCherry mitochondrial signals. Arrows highlight mCherry‐only signal co‐localising with LAMP1, a marker for lysosomes, confirming mitophagy. Scale bar, 5 μm. For details see McWilliams *et al*. 121.

Outside of the nervous system, renal tubules exhibit a high metabolic demand. This requirement is in large part to drive the massive salt and protein reabsorption from the filtrate. As a consequence of this, these postmitotic tubular cells are second only to heart cardiomyocytes in terms of their mitochondrial content 127. It is, therefore, not surprising that impaired mitophagy has been postulated to contribute to a range of renal pathologies, including acute kidney injury 128, 129. It should not be overlooked that exactly 60 years ago, scientists described perhaps the first instances of mitophagy by observing degraded mitochondria within lysosome‐like structures in rabbit kidney tubules 130. In wild‐type *mito*‐QC animals, a striking level of mitophagy was observed throughout the cortex of the adult kidney (see Fig. 3 and 121). However, a clear difference in the degree of mitophagy was observed between distinct tubules of the nephron. Proximal convoluted tubules (PCTs) exhibited a major enrichment in mitophagy compared with distal convoluted tubules (DCTs), even though both types of tubule are loaded with mitochondria. This discovery highlighted another important conceptual advance: that the degree of mitophagy within a cell is independent from its mitochondrial mass. What then might account for the heterogeneity of this basal mitophagy observed *in vivo*? Metabolic context is likely to play important role. For example, differences in adult renal mitophagy could be a function of altered metabolic states between tubules and/or functional demand that requires a constant turnover of mitochondria. Indeed, oxygen consumption is believed to be greater in PCTs versus DCTs 131. It could also be that PCT mitochondria are more vulnerable to mitochondrial stress than their DCT counterparts, although this is highly speculative and the actual comparative mitochondrial biology between these structures remains to be elaborated in greater mechanistic detail. Nonetheless, comparative mitochondrial differences between tubules have been described. For example, mitochondrial membrane potential is reported to be different between DCTs and PCTs 132. The same study reports higher ROS production in PCTs than DCTs. Taken together, these collective observations highlight the need for a deeper understanding of the interplay between mitochondrial metabolism and mitophagy within different cell subtypes *in vivo*.

The selective nature of autophagy *in vivo* is also highlighted when evaluating *mito*‐QC data in the context of pioneering studies by Mizushima on *in vivo* autophagy 133. Elegant work using a general autophagy reporter (GFP‐LC3) mouse model showed elevated LC3‐positive autophagosomes in adult kidney glomeruli, indicating elevated general autophagy in this renal structure. However, in *mito*‐QC animals, low levels of mitophagy were observed in adult glomeruli compared with tubules. Unexpectedly, during embryonic development, this distinct renal mitophagy is reversed, with elevated mitochondrial turnover occurring in glomeruli but not tubules 121. Therefore, not only are the physiological instances of autophagy highly context dependent but also demonstrate remarkable specificity, in this case for mitophagy.

### Mitophagy modalities: intracellular and extracellular destruction

Autophagy has been classically studied as an intracellular, self‐catabolic quality control pathway. However, it is emerging that this assumption may not be entirely accurate. Davis *et al*. 134 reported the trans‐cellular elimination of mitochondria *in vivo*. They observed that neuronal mitochondria targeted for elimination were expelled from axonal evulsions within the optic nerve head and degraded by neighbouring glial cells. It will be important to understand and further elaborate how ‘axonal‐trans mitophagy’ and other extracellular mitochondrial trafficking mechanisms contribute to mitochondrial homeostasis *in vivo*. These data challenge our assumptions of mitochondrial quality control pathways as being intrinsic to a particular cell and highlight the importance of studying mitochondrial homeostasis in the context of the neurogliovascular unit – a fundamental entity of the nervous system *in vivo* 54.

## The future

Taken together, these discoveries demonstrate that further research is required to understand the orchestration of metabolism and mitophagy and their contribution to tissue development and long‐term function. The molecular regulation of these processes remains enigmatic. It is, thus, accurate and appropriate to conclude that *in vivo* mitophagy remains a complex, context‐dependent process: the next frontier of mitophagy research.

## Author contributions

CER, TGM and IGG wrote the review.
